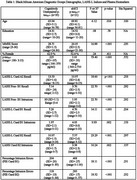# Plasma Biomarkers in Diverse Populations

**DOI:** 10.1002/alz70856_101158

**Published:** 2025-12-25

**Authors:** David A. Loewenstein

**Affiliations:** ^1^ University of Miami School of Medicine, Miami, FL, USA

## Abstract

**Background:**

Recent advancements in plasma biomarkers, particularly *p*‐tau217, have highlighted its potential for early Alzheimer's Disease (AD) detection. *p*‐tau217 demonstrates diagnostic accuracy comparable to amyloid PET and cerebrospinal fluid (CSF) tests, with strong sensitivity and specificity to Aβ PET and tau PET results. This biomarker correlates with both current AD brain pathology and future cognitive decline, making it valuable for tracking disease progression. Although *p*‐tau217 shows promise as a predictor for early AD detection, further research is needed to validate its utility in diverse populations before it can be established as a routine diagnostic tool.

**Method:**

We conducted comprehensive clinical and neuropsychological evaluations of 96 Spanish‐speaking Hispanic/Latino (H/L) and 56 Black/African American (B/AA) older adults. Participants underwent MRI, amyloid PET scans, and plasma biomarker testing, including *p*‐tau217. A stepwise binary logistic regression analysis was performed to assess how well a cognitive challenge test could differentiate between cognitively unimpaired (CU) *p*‐tau217‐ and amnestic mild cognitive impairment (aMCI) *p*‐tau217+ B/AA individuals.

**Results:**

Receiver operating characteristic (ROC) curve analyses using amyloid PET as the gold standard revealed a strong discriminative ability with an area under the curve of 0.89 (*p* < .001) for *p*‐tau217 levels measured by SIMOA (Alzpath). Applying Youden's index, we identified a *p*‐tau217 cut‐off of 0.55 pg/ml that provided an optimal balance of sensitivity (85%) and specificity (87%). This cut‐off was consistent across both H/L and B/AA groups. Cognitive challenge tests assessing proactive semantic interference (PSI) and intrusion errors were the best predictors of CU *p*‐tau217‐ versus aMCI *p*‐tau217+, with high sensitivity (80%) and specificity (91.7%).

**Conclusions:**

A *p*‐tau217 cut‐off of 0.55 pg/ml aligns well with positive Aβ PET scans, indicating underlying AD pathology, and provides an effective balance of sensitivity and specificity in a multicultural cohort. Additionally, cognitive challenge tests may enhance diagnostic accuracy, offering scalable potential for early AD detection in underserved populations.